# Site-Specific Isopeptide Bond Formation: A Powerful Tool for the Generation of Potent and Nontoxic Antimicrobial Peptides

**DOI:** 10.1021/acs.jmedchem.2c00061

**Published:** 2022-03-15

**Authors:** Naiem Ahmad Wani, Elad Stolovicki, Daniel Ben Hur, Yechiel Shai

**Affiliations:** Department of Biomolecular Sciences, Weizmann Institute of Science, Rehovot 76100, Israel

## Abstract

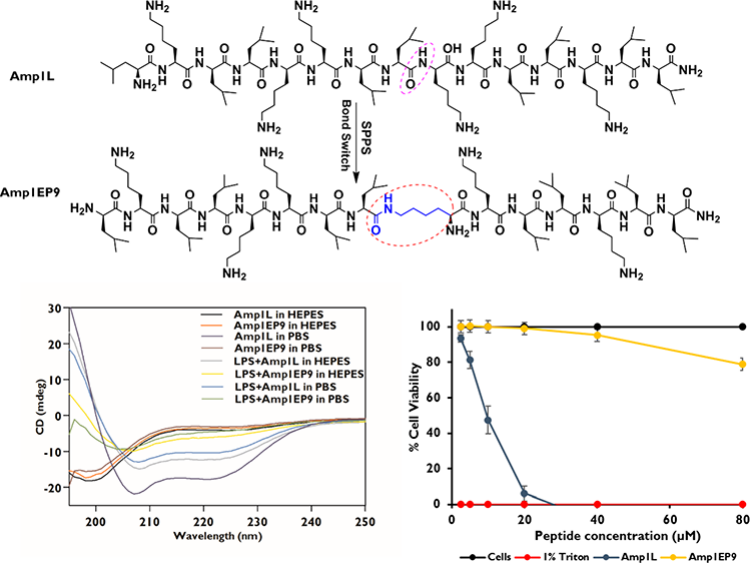

Antimicrobial peptides (AMPs) have the potential to treat multidrug-resistant bacterial infections. However, the clinical application of AMPs is prevented by their toxicity and poor proteolytic stability. Here, a site-specific approach is used to generate new AMPs to improve their efficacy against bacterial pathogens while reducing their toxicity. We modified and generated a new series of antimicrobial peptides from the leucine- and lysine-rich antimicrobial peptide Amp1L (LKLLKKLLKKLLKLL) by the site-specific incorporation of an isopeptide bond while retaining the peptide’s size, sequence, charge, and molecular weight. This single bond switch provides the peptides with a weak helical conformation, strong antimicrobial activity, resistance to proteolytic degradation, low toxicity, and lower hemolytic activity. This new site-specific approach offers a powerful tool for developing potent and nontoxic antimicrobial drugs.

## Introduction

One major health concern is the global spread of multidrug-resistant (MDR) pathogenic bacteria.^1^ New antibiotics are urgently required to combat resistant pathogenic micro-organisms. Antimicrobial peptides (AMPs) are produced by many organisms as part of their defense strategy against microbes.^2^ AMPs have drawn much attention as potential antibiotics against multidrug-resistant bacteria due to their potent and broad-spectrum activities.^3−7^ However, natural AMPs are toxic, proteolytically unstable, and hemolytic and thus cannot be used as drugs.^8−10^ Several approaches were suggested to overcome these hurdles, such as incorporating unnatural amino acids, substituting l-proteinogenic amino acids with d-proteinogenic amino acids, N-terminal acetylation, C-terminal amidation, cylization, lipidation, and replacing amino acids.^11−16^ A new approach to modulate AMPs uses an isopeptide bond replacement.^17^ The isopeptide bond is an amide bond between the carboxyl group of one amino acid and the epsilon amino group of another amino acid. The isopeptide bond is a natural bond that appears in HK97 bacteriophage capsid formation, Gram-positive bacterial pili, and ubiquitin in humans.^18−20^

Our previous study generated two new antimicrobial peptides with three isopeptide bonds, named Amp1EP and MSIEP, from the well-known antimicrobial peptides Amp1L and MSI-78 (pexiganan), respectively.^21,22^ Incorporating three isopeptide bonds improves the peptide’s stability and reduces both the toxicity and hemolytic activity but also reduces the antimicrobial activity.^17^ In this work, we systematically introduced a single isopeptide bond and examined the effect of the bond switch on the biological and biophysical properties of the peptides. Starting from the parental peptide Amp1L,^14,23^ we generated six new peptides named Amp1EP2, Amp1EP5, Amp1EP6, Amp1EP9, Amp1EP10, and Amp1EP13. Each of these peptides was synthesized by placing a single isopeptide bond in one of its lysine residues while retaining properties such as size, sequence, charge, and molecular weight. The antibacterial activity of all these peptides was examined against a panel of two Gram-negative bacteria (*Pseudomonas aeruginosa* PA01 and *Escherichia coli* K12 parental type) and two Gram-positive bacteria (*Staphylococcus aureus* ATCC 6538P and *Bacillus subtilis A* TCC 6051). Interestingly, all the new peptides are more potent against all the bacteria strains tested than the parental peptide. Moreover, of all the peptides tested, Amp1EP9 is less toxic toward human red blood cells (hRBCs), murine macrophage cells (RAW 264.7), and human embryonic kidney cells (HEK 293). Furthermore, Amp1EP9 has significantly better protection from proteolytic degradation. Altogether, we show a new approach for a site-specific isopeptide bond switch that can be applied to modulate known potent AMPs into useful drugs.

## Results

**Figure 1:**
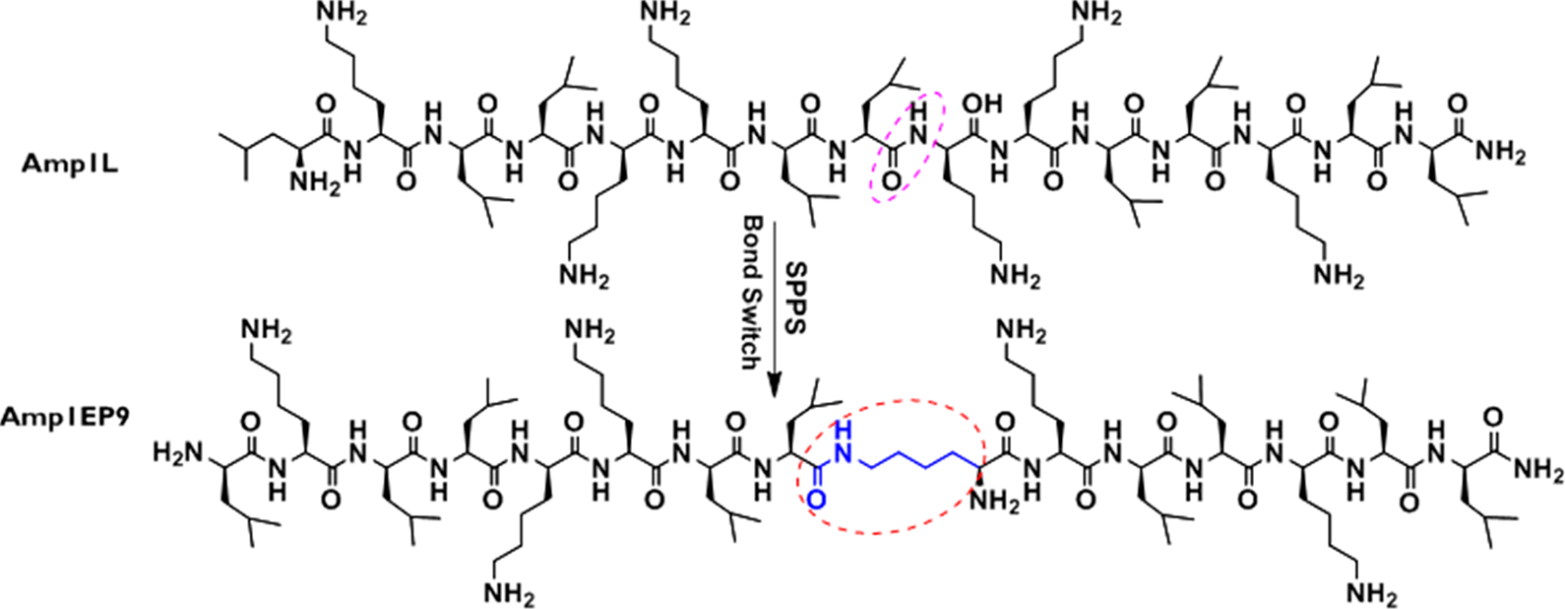
Chemical structures of peptides Amp1L and Amp1EP9.

In this work, a site-specific approach was applied to synthesize a series of six peptide analogs of the synthetic antimicrobial peptide Amp1L. Each new peptide was generated by switching the peptide bond in one of Amp1L’s lysine residues with an isopeptide bond (Table 1). The chemical structures of the peptides Amp1L and Amp1EP9 are shown in Figure 1.

**Table 1:** Designations, Sequences, and Relative Hydrophobicities of Peptides^a^

peptide	sequence^b^	relative hydrophobicity^c^ (% AcN)	retention time^d^ (min)
Amp1L	LKLLKKLLKKLLKLL	69.4	29.7
Amp1EP2	L**K**LLKKLLKKLLKLL	57.8	23.9
Amp1EP5	LKLL**K**KLLKKLLKLL	57	23.5
Amp1EP6	LKLLK**K**LLKKLLKLL	56.8	23.4
Amp1EP9	LKLLKKLL**K**KLLKLL	53.8	21.9
Amp1EP10	LKLLKKLLK**K**LLKLL	54.2	22.1
Amp1EP13	LKLLKKLLKKLL**K**LL	62.4	26.2

a Underlined and bolded lysine K2, K5, K6, K9, K10, and K13 in all peptides are involved in isopeptide bond formation.

b All of the peptides are amidated at their C-termini and have same charge, length, and molecular weight.

c Relative hydrophobicity is reflected by the percent of acetonitrile at the retention time.

d Reversed-phase HPLC retention time in the C18 column using a gradient of 10–90% acetonitrile in water for 40 min.

### Peptide Toxicity

**Figure 2:**
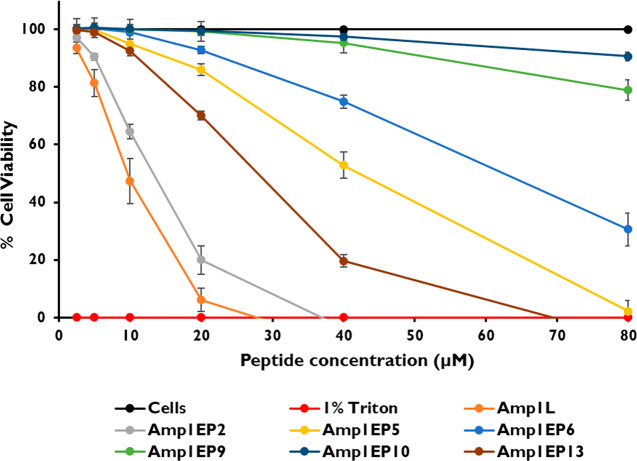
Hemolytic activity of the peptides (2.5–80 μM) on hRBCs. Untreated cells were used as a negative control, and cells treated with 1% Triton X-100 were used as a positive control. All data represent the mean ± SD from three biological repeats performed in duplicate. The cell viability is plotted (100% – hemolysis). One-way analysis of variance was used to analyze the data. Results show a statistically significant difference (*p* < 0.005).

Key condition for using AMPs in medical applications is a low toxicity toward mammalian cells. Therefore, we studied the toxicity of this class of new peptides to hRBCs. Due to direct membrane lysis, Amp1L showed strong toxicity to human erythrocytes at 12.5 μM. In contrast, Amp1EP9 and Amp1EP10 were not toxic at 80 μM, the highest concentration tested. Moreover, the other peptides Amp1EP6, Amp1EP5, Amp1EP13, and Amp1EP2 had reduced toxicities compared to that of the parental peptide Amp1L (Figure 2).

**Figure 3:**
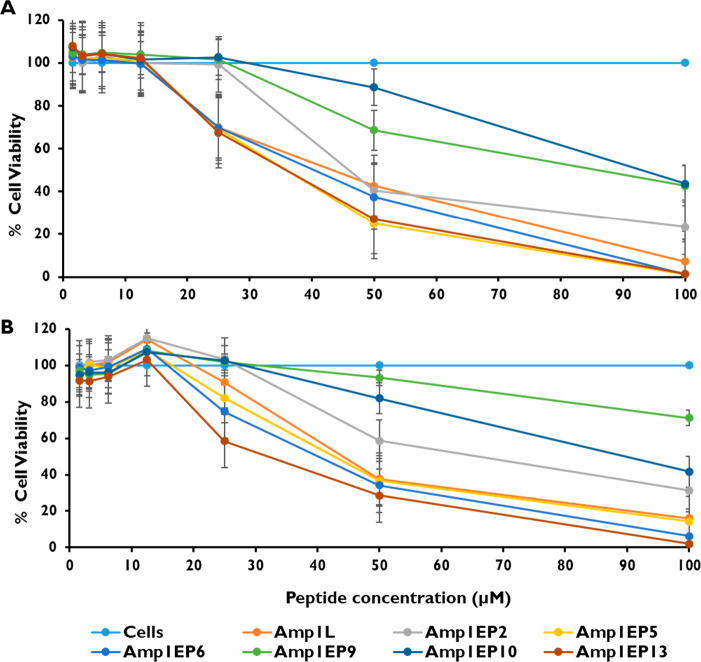
Cytotoxicity of peptides (1.56–100 μM) on (A) RAW 264.7 and (B) HEK 293 cells. Cell viability was analyzed and quantified by measuring the absorption at 450 nm. The data are presented as the mean percent viability. All data represent the mean ± SD from three biological repeats performed in duplicate. One-way analysis of variance was used to analyze the data. Results show a statistically significant difference (*p* < 0.001).

Subsequently, we evaluated the toxicity of peptides against two types of mammalian cell lines, RAW 264.7 and HEK 293 cells, using the XTT dye reduction assay. The cytotoxicity was tested in the peptide concentration range between 1.56 and 100 μM. Among all the peptides tested, Amp1EP9 and Amp1EP10 were the least toxic, and reduced the viability of RAW 264.7 cells by only 31% and 12%, respectively, at 50 μM. Moreover, both Amp1EP9 and Amp1EP10 reduced the viability by 56% at 100 μM, the highest concentration tested. Similarly, in the case of HEK 293 cells at 50 μM, Amp1EP9 and Amp1EP10 reduced the cell viability by only 7% and 20%, respectively, whereas the peptide Amp1EP9 reduced the cell viability by 29% and Amp1EP10 reduced the cell viability by 59% at 100 μM, the highest concentration tested. In contrast, the parental peptide Amp1L and other modified peptides were relatively toxic at these concentrations (Figure 3A and B). These results are in agreement with hemolysis tests where these peptides were shown to be less toxic to human erythrocytes, confirming that their alternative mode of membrane action leads to improved safety profiles.

### Peptide Structure

**Figure 4:**
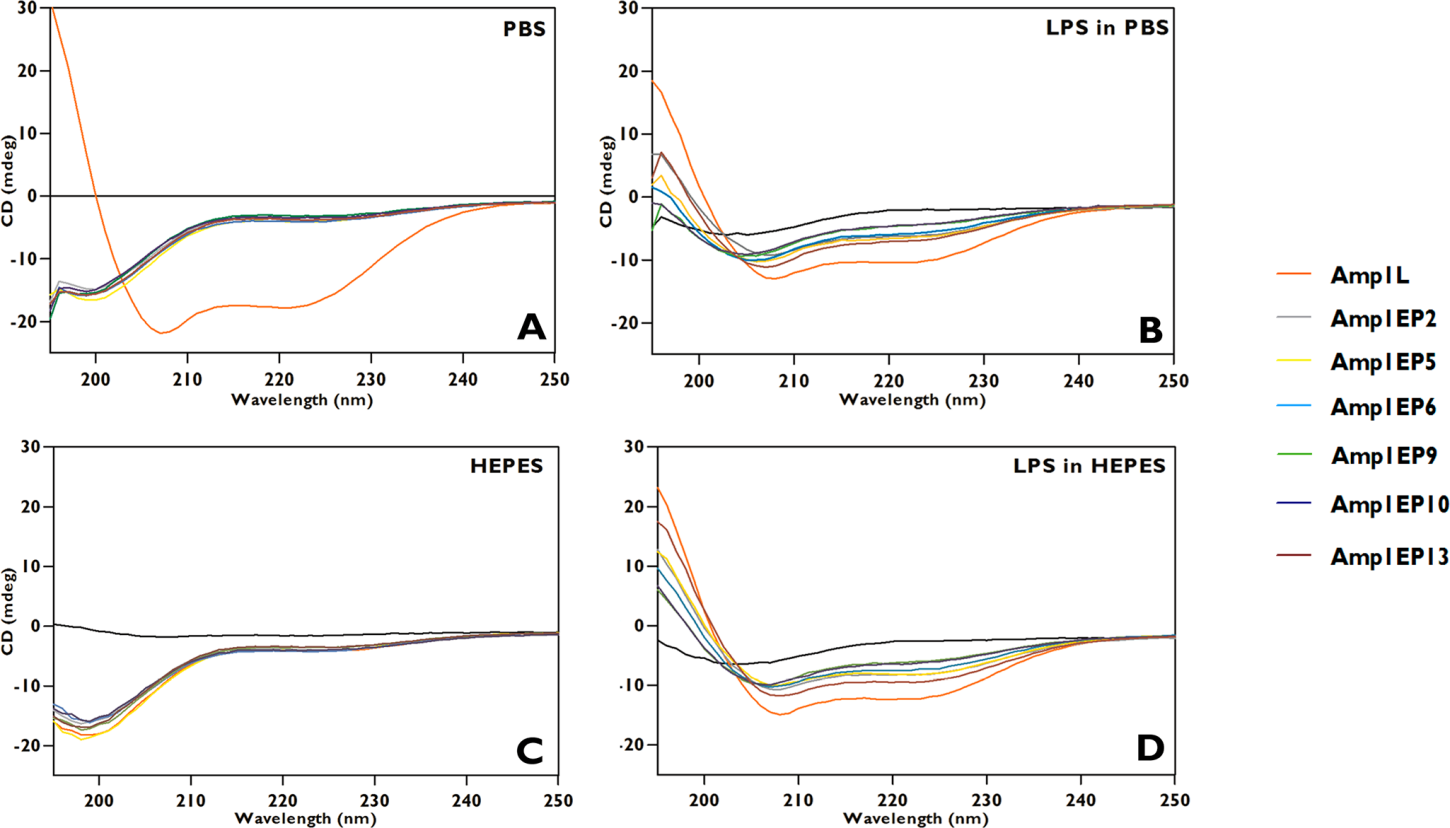
Secondary structures of the peptides. Circular dichroism spectra of the peptides were obtained in (A) PBS (pH 7.4), (B) LPS/PBS, (C) HEPES (pH 7.2) and (D) LPS/HEPES.

The secondary structures of all the peptides were analyzed by circular dichroism (CD) in solution in the range of 195–250 nm. The results revealed that all new peptides had random coil structures at a 50 μM concentration in PBS (pH 7.4) and HEPES (pH 7.2) as opposed to Amp1L, which displayed a helical structure in PBS. Further, with the addition of lipopolysaccharide (LPS) (1:1 ratio, LPS/peptide) all the new peptides had varying levels of weak α-helical conformations in contrast to Amp1L, which adopted a well-ordered helical structure in both PBS and HEPES buffers as indicated by the maximum at 195 nm and the double minima at 208 and 222 nm (Figure 4). Indeed, the estimation of the secondary structures of the peptides, using CDNN software, predicts only 38.1% and 52.5% α-helical contents for Amp1EP9 in LPS with PBS and HEPES, respectively, in contrast to 80.2% and 90.2% for the parental peptide Amp1L. Thus, the insertion of the isopeptide bond disrupts the α-helical conformation of the peptide regardless of its position. However, the isopeptide bond near the N- or C-terminus of the peptide has less effect on the conformation than the isopeptide bond near the center of the peptide, (e.g., Amp1EP9 and Amp1EP10) (Table 2). Interestingly, the results show that a peptide becomes less and less toxic to hRBC as its α-helical conformation becomes more and more disrupted (Figures 2 and 4).

**Table 2:** Predictions for the Secondary Structure of the Peptides by the CDNN Analysis Software

	LPS in PBS					LPS in HEPES				
	α-helix	antiparallel β-sheet	parallel β-sheet	β-turn	random coil	α-helix	antiparallel β-sheet	parallel β-sheet	β-turn	random coil
Amp1L	80.8	0.2	2.0	11.2	5.8	90.2	0.1	1.0	9.7	2.2
Amp1EP2	53.5	4.0	3.8	16.2	10.5	68.8	1.0	2.7	13.6	7.0
Amp1EP5	50.3	7.2	3.6	17.6	8.4	66.9	1.1	2.9	13.6	8.3
Amp1EP6	45.8	10.9	3.9	18.6	9.0	62.0	2.2	3	15	7.7
Amp1EP9	38.1	19.1	4.7	20.0	10.9	52.5	5.4	3.6	16.9	8.9
Amp1EP10	36.9	20.2	4.9	20.2	11.6	53.6	4.9	3.5	16.7	8.6
Amp1EP13	58.5	3.7	3	16.3	6.9	79.3	0.3	2.0	11.7	5.2

### Proteolytic Resistance

**Figure 5:**
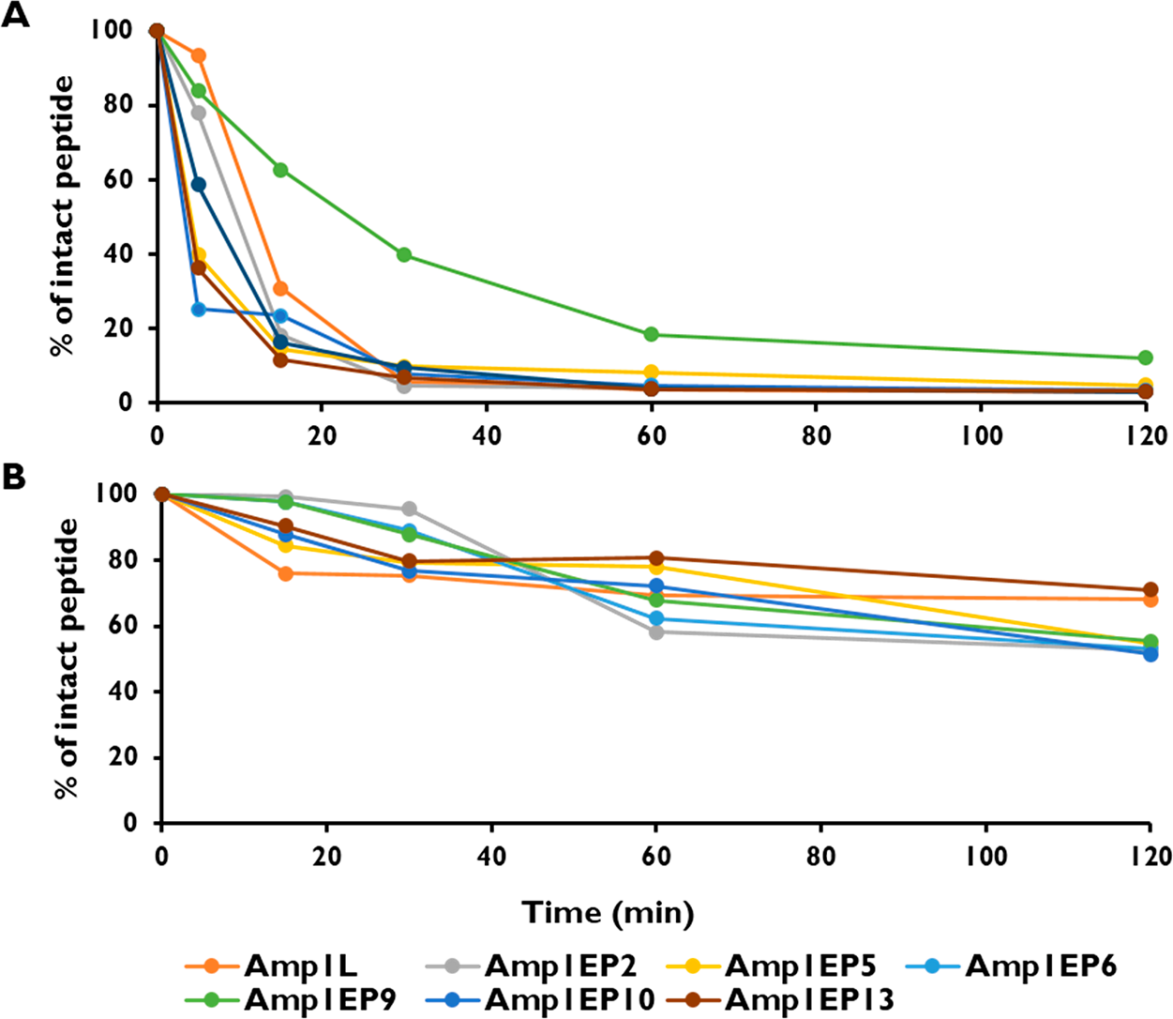
Resistance of peptides to (A) trypsin digestion and (B) human serum. Percentages of intact peptides were determined by reversed-phase HPLC comparative to the peak areas acquired at *t*_0_ (control at 0 min set to 100% for each peak).

One hindrance to AMPs reaching clinical application is their poor resistance against proteolytic degradation *in vivo.*(24) To test the proteolytic resistance, the new peptides were incubated with trypsin (10 μg/mL) for different time intervals. After 30 min of incubation, 40% of peptide Amp1EP9 remained intact. In contrast, neither the parental peptide Amp1L nor any of the other modified peptides were detected after 30 min. These results demonstrated that the site-specific isopeptide bond switch is crucial to improving the proteolytic stability (Figure 5A). Furthermore, the stability of the peptides was assessed by incubating them in human plasma (10% v/v) rich in proteases thrombin and plasmin for different times at 37 °C, which revealed that the parental peptide and the peptides incorporating the isopeptide bond were stable after 2 h. (Figure 5B).

### Antibacterial Activity

The antibacterial activity of all the peptides was tested against two Gram-negative (*P. aeruginosa* and *E. coli*) and two Gram-positive (*S. aureus* and *B. subtilis)* bacterial strains. All the peptides showed appreciable antimicrobial activity against all the bacteria tested. Interestingly, the new peptides with a single isopeptide bond were more potent than the parental peptide Amp1L, as shown from the minimum inhibitory concentration (MIC) experiments. The MICs of new peptides range from 1.56 to 6.25 μM compared to the parental peptide Amp1L whose MIC is 12.5 μM (Table 3).

**Table 3:** Antibacterial Activities of Peptides

	MIC (μM)						
bacteria strain	Amp1L	Amp1EP2	Amp1EP5	Amp1EP6	Amp1EP9	Amp1EP10	Amp1EP13
*P. aeruginosa*	12.5	1.56	3.12	1.56	3.12	3.12	1.56
*E. coli*	12.5	1.56	1.56	1.56	3.12	3.12	1.56
*S. aureus*	12.5	1.56	3.12	1.56	6.25	3.12	1.56
*B. subtilis*	12.5	1.56	1.56	1.56	3.12	3.12	1.56

### Investigation of Membrane Permeability

**Figure 6:**
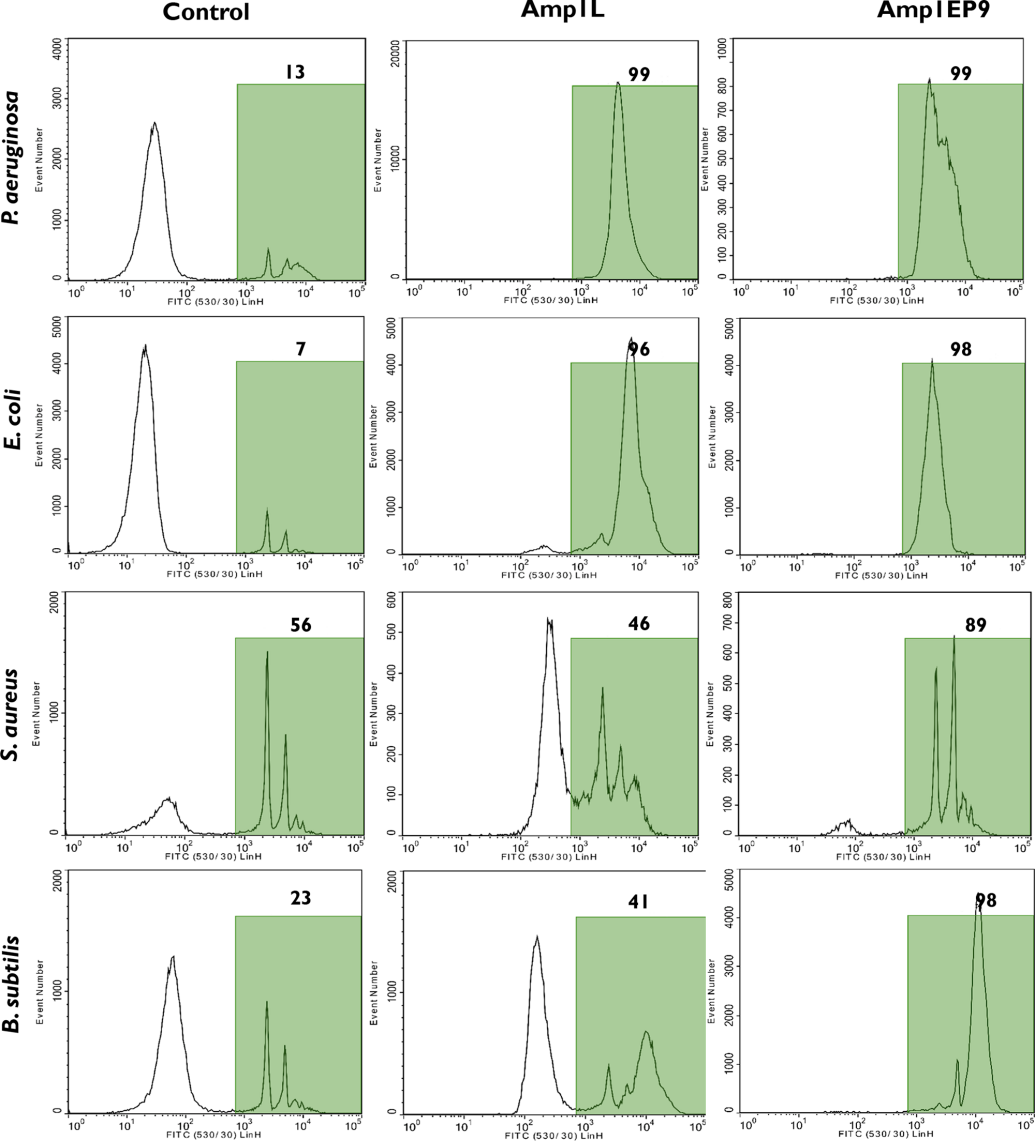
Membrane permeability in bacterial cells treated with the peptides at 1× their MICs. Flow cytometry was used to determine the fluorescent intensity of SYTOX-Green. The histograms shows the distribution of the green fluorescence intensity for all the cells that were positively labeled with FM 4-64 dye.

Based on the above results, Amp1EP9 has a low toxicity, a high stability, and potent antimicrobial activity and therefore was chosen for a membrane permeabilization study by flow cytometry and confocal fluorescence microscopy. To measure the permeability of the bacteria membrane, SYTOX-Green dye was used. SYTOX-Green is a cell-impermeable nucleic acid stain that only labels bacteria with compromised plasma membranes, and its fluorescence signal increases >500-fold when binding to DNA. It enabled us to follow the dynamics of the puncturing of the bacterial membrane by the peptide. Since bacteria are small, their light scattering pattern partly overlaps with scatter signals from foreign particles in the sheath fluid and other flow cytometer noise sources. Therefore, the bacterial membrane dye FM 4-64 was is used to help discriminate bacteria cells from noise, thus improving the measurement accuracy of the percentage of cells affected by the peptide. First, the bacterial suspension was labeled with FM 4-64 and SYTOX-Green (control). Next, the peptides Amp1L and Amp1EP9 were added at their MIC concentrations, and the suspensions were measured in a flow cytometer. Both the peptides permeabilized the membrane of all the bacteria tested. Treatment with both Amp1L and Amp1EP9 against the Gram-negative bacteria *P. aeruginosa* and *E. coli* produced 98% positively green fluorescence. Moreover, Amp1EP9 permeabilized 98% and 89% of the Gram-positive *B. subtilis and S. aureus*, respectively. In contrast, Amp1L permeabilized only 41% and 46% of these bacteria cells. Note that for *S. aureus* 56% of the cells were already positively green before the peptide was added. This is a result of the effect of Amp1L on the scattering pattern from the bacteria. Upon treatment with Amp1L, the bacteria scatter moved out of their gate on the side and forward scatter plot. Furthermore, many bacteria lost the red fluorescence signal from FM 4-64 membrane dye. These signal changes make it hard to determine bacteria cells from noise and thus decreased the accuracy of determining the effect of the peptide. In contrast, Amp1EP9 does not cause similar effects on most bacteria (Figure 6).

**Figure 7:**
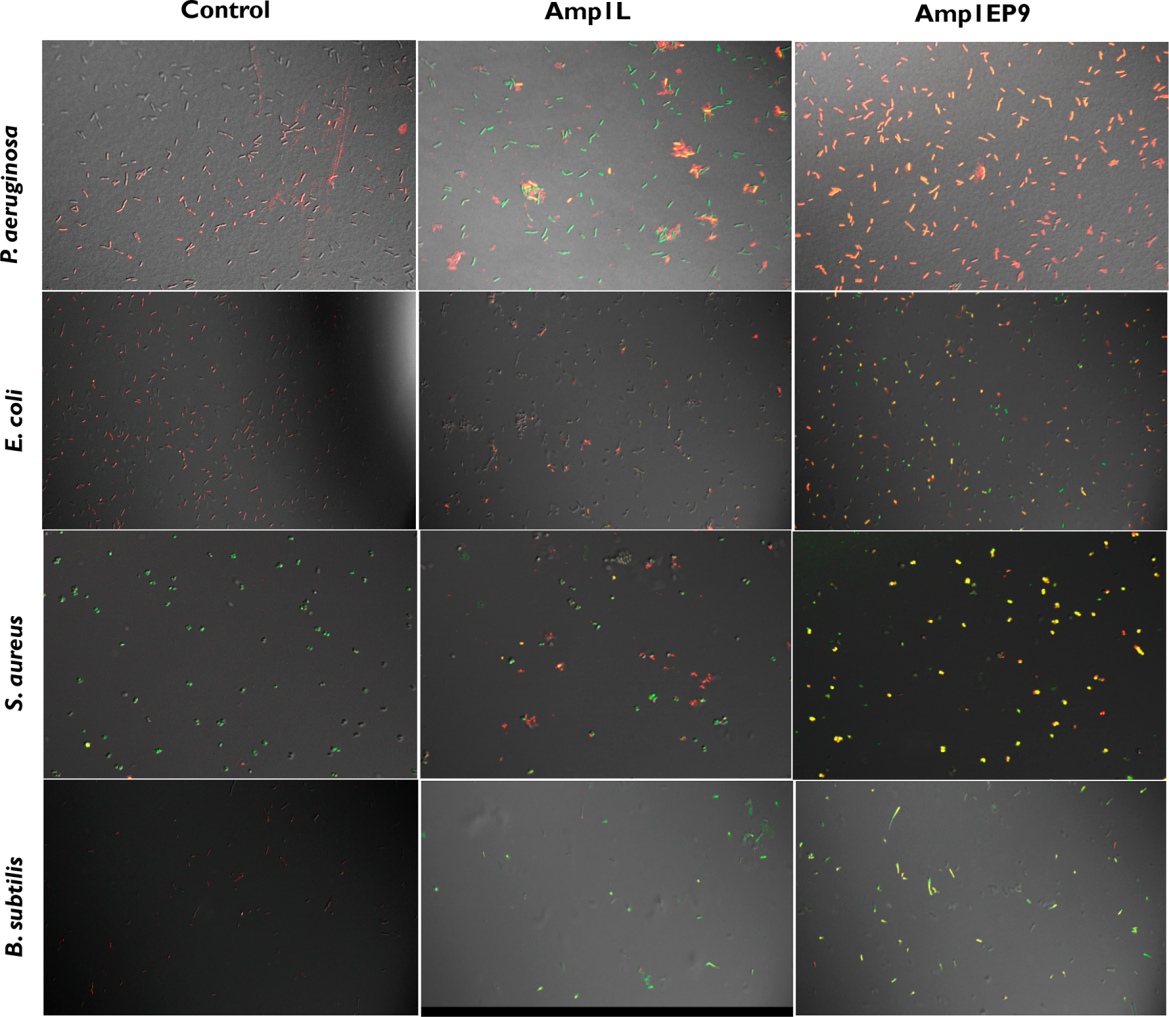
Confocal fluorescence microscopic images of bacteria treated with FM 4-64 and SYTOX-Green-labeled Amp1L and Amp1EP9 at 1× their MICs suggested that Amp1EP9 was able to bind and disrupt the cell membrane effectively.

We investigated the disruption of bacterial membranes by Amp1L and Amp1EP9 using confocal microscopy. In this experiment, we labeled the bacterial membrane with the lipophilic dye FM 4-64 and the DNA dye SYTOX-Green and examined the morphology of bacteria using a confocal microscope in the absence and presence of either Amp1L or Amp1EP9 peptides. The penetration of SYTOX-Green to all the bacteria were observed. Bacteria treated with Amp1EP9 showed morphologies similar to those of untreated bacteria on all the bacteria strains. Most of the cells were green, indicating that the membrane integrity was compromised in agreement with the flow cytometry data. The bacteria treated with the parental peptide Amp1L have less uptake of SYTOX-Green, indicating less membrane disruption. Interestingly, Amp1L treatment leads to bacteria aggregation (Figure 7).

## Discussion

The urge to eradicate multidrug-resistant bacterial infections powered the use of AMPs as an attractive strategy with high structural diversity and broad-spectrum antimicrobial activity.^25−27^ Rational design approaches have been used to generate synthetic analogs of antimicrobial peptides, reducing the limitations and increasing advantages.^1,11^ However, several AMPs have failed in phase II and III testing^28^ because AMPs are vulnerable to proteolysis and have toxicity associated with mammalian membrane lysis.^29^ We have shown a new strategy that improved the antimicrobial peptide analogs. In this work, the modified AMPs were generated using a site-specific approach by replacing a peptide bond with an isopeptide bond at various positions in the lysine- and leucine-rich 15-mer peptide Amp1L at six lysine positions. Mutation to any one of these lysine’s leads to dramatically changed physicochemical behaviors. Its short sequence, simple amino acids, and potent cytotoxicity made it a model AMP to investigate basic principles that govern the biophysical properties and biological activity of AMPs. The toxicity potential was evaluated against human erythrocytes, RAW 267.9 cells, and HEK 293 cells (Figure 2 and 3A and B, respectively). Peptide Amp1EP9 displayed a lower toxicity than the parental peptide, showing the safety of peptide and highlighting its potential for clinical development. Structural characterization studies of the peptides were done using CD in PBS and HEPES, contributing to the understanding that all peptides had random coils except the Amp1L in PBS. On contact with the LPS micelles, the new AMPs exhibited weak α-helical conformations as observed by circular dichroism (Table 2 and Figure 4). This unveiled that switching the peptide bond to an isopeptide bond near the center of the peptide (e.g., Amp1EP9 and Amp1EP10) was potent and nontoxic to the mammalian cells.

We investigated the disruption of the bacterial membrane using flow cytometry and confocal microscopy and demonstrated that the peptide with the single bond switch (Amp1EP9) achieved efficient cell membrane disruption compared to the parental peptide. In addition, the insertion of a single isopeptide bond conferred near protection from proteolytic sensitivity and plasma instability (Figure 5A and B).

Interestingly, the new peptides are more potent than the parental peptide even though they have less helical conformations. This may be due to the changes in physiochemical properties such as aggregation, oligomerization, and penetration. Further study of their mode of action will be done soon.

Altogether, by all parameters, including efficacy against bacteria, low toxicity, and stability in human surroundings (trypsin and blood serum), Amp1EP9 is the leading AMP compared to all AMPs tested. This new strategy of switching an isopeptide bond at one specific position in AMPs leads us to learn how to improve lytic, toxic, ineffective, and unstable AMPs. This investigation advances our understanding of the parameters of new AMPs and encourages us to develop new antimicrobial treatments. In the future, it will be interesting to apply this method to reduce the toxicity of very potent peptides, thus transforming them into useful drugs. A further direction for the research is to incorporate isopeptide bonds of other amino acids in AMPs.

## Conclusions

In conclusion, this site-selective isopeptide bond switch serves as a proof of principle for the versatility and potentiality of fine-tuning AMP biological activity. Screening the antimicrobial activity, serum susceptibility, and hemolysis of human erythrocytes against the synthesized peptides unveiled that a single isopeptide bond switch could provide antimicrobial peptides with potent antimicrobial activity, reduced toxicity, and high proteolytic stability. A primary antimicrobial mechanism study showed that Amp1EP9 had bacterial killing kinetics by disrupting the cell membrane. This approach can provide an important tool and has great potential for developing potent and nontoxic antimicrobial drugs.

## Experimental Section

### Chemicals

All the reagents for the synthesis of the peptides were obtained from commercial sources and used without further purification. The amino acids Fmoc-Leu-OH, Fmoc-Lys(Boc)-OH, and Boc-Lys(Fmoc)-OH) and coupling reagents (*N*,*N*′-diisopropylcarbodiimide (DIC) and ethyl cyanohydroxyiminoacetate (oxyma)) were purchased from Novabiochem. Trifluoroacetic acid (TFA), piperidine, and triisopropyl silane (TIS) were purchased from Sigma-Aldrich. Solvents for peptide synthesis, purification, and analysis, including *N*,*N* dimethylformamide (DMF), dichloromethane (DCM), diethyl ether (Et_2_O), and acetonitrile (MeCN), were obtained from Bio-Lab Ltd. The rink amide resin (0.57 mmol/g) was purchased from Iris Biotech Gmbh, and the manufacturer’s reported loading was used to caculate the yields of the final product.

### Peptide Synthesis and Cleavage

The peptide synthesis was carried out on Rink Amide MBHA resin by using the Fmoc strategy on a Liberty Blue peptide synthesizer (CEM, Matthews, NC) as reported earlier.^30^ To achieve the synthesis of new peptides from the parental peptide Amp1L, we replaced all the six lysines one by one to generate six new peptides. Boc-Lys(Fmoc)-OH was used to make an isopeptide bond, which is formed between a carboxyl group of one amino acid and epsilon amino group of another amino acid, rather than Fmoc-Lys(Boc)-OH, which is used to make regular peptide bonds. The positions involved of isopeptide bond formation in the new peptides are K2, K5, K6, K9, K10, and K13. The resin-bound peptide was washed thoroughly with DMF and then DCM, dried, and cleaved. Cleavage was done using 95% TFA, 2.5% water, and 2.5% TIS for 120 min at room temperature. The crude peptides were washed from the resin using TFA, precipitated using cold diethyl ether, and air dried.

### Peptide Purification

The purification was done by using reversed-phase high-performance liquid chromatography (RP-HPLC) on a Agilent Technologies 1200 Series instrument with a reversed-phase C4 column (5 μm particle size, 250 × 10 mm) at a flow rate of 1.8 mL/min and was monitored with a UV detector at 215 nm. Linear gradients of 10–90% acetonitrile in water containing 0.1% TFA were used for peptide purification. Final products were obtained by freeze-drying the collected pure fractions.

### HPLC Analysis

The purities of all peptides were analyzed on an Agilent Technologies 1260 Infinity spectrometer with a C18 reversed-phase column (Bio-Rad analytical column, 250 × 4 mm, 300-Å pore size, 7 μm particle size) with a flow rate of 0.6 mL/min using a gradient of 10–90% acetonitrile in water both containing 0.1% TFA (v/v) for 40 min with UV detection at 215 nm. The molecular masses of all the peptides were determined by TOF-MS. The purity of all peptides examined for biological activity was >95%

### Hemolytic Activity

Human red blood cells (hRBCs) were used to measure the hemolytic effect of the peptides by measuring the amount of hemoglobin released after treatment.^31^ Fresh human blood was obtained from healthy volunteers and processed by centrifugation at 600 × *g* for 5 min to obtain RBCs. The plasma was removed, and the lower layer containing RBCs was washed three times in sterile phosphate-buffered saline (PBS, pH 7.4) and centrifuged at 600 × *g* for 5 min. The purified hRBCs were diluted in PBS to a final concentration of 2% (v/v), then 100 μL of the hRBC suspension was incubated with 100 μL of different concentrations (2.5–80 μM) of a peptide dissolved in PBS. After 1 h of incubation at 37 °C under 5% CO_2_, intact hRBCs were pelleted by centrifugation at 600 × *g* for 5 min, then the supernatant was taken out and transferred to another 96-well plate. The sample supernatant absorbance was measured at 450 nm using a microplate auto reader (SynersyMx, Biotek). Untreated cells were used as a negative control, and cells treated with 1% Triton X-100 were used as a positive control. The percentage of hemolysis was calculated as [(sample absorbance – negative control absorbance)/(positive control absorbance – negative control absorbance)] × 100.

### Cell Survival Assay

The cytotoxicity of the peptides toward RAW 264.7 and HEK 293 cells was assayed using the colorimetric 2,3-bis(2-methoxy-4-nitro-5-sulfophenyl)-2*H*-tetrazolium-5-carboxanilide (XTT) method. Here, 1 × 10^5^ cells per well were incubated with serially diluted peptides in concentrations ranging from 1.5 to 100 μM for 24 h at 37 °C in 5% CO_2_. Moreover, the last two columns with media served as the blank, and the cells plus media served as the 100% survival control. After incubation, the cell viability was assessed by the XTT reaction solution (100 μL), and benzene sulfonic acid hydrate and *N*-methyl dibenzopyrazine methyl sulfate mixed in a 50:1 ratio was added for an additional 4 h at 37 °C. The absorbance at 450 nm was then measured using a microplate auto reader (SynersyMx, Biotek). After the deduction of the blank read, the cell viability was calculated relative to the 100% survival control.

### Circular Dichroism

The secondary structure of the peptides was examined using Chirasca CD Spectrometer (Applied Photophysics Ltd., Jasco, Tokyo, Japan) at 25 °C using a thermostatic quartz cuvette with a path length of 1 mm and analyzed for structure proportions using CDNN.^32^ Peptides were dissolved in PBS (pH 7.4) and 5 mM HEPES (pH 7.2) to a final concentration of 50 μM in the presence or absence of 50 μM purified *P. aeruginosa* lipopolysaccharide (LPS) (Sigma-Aldrich). Each spectrum was recorded from 195 to 250 nm at a 1 nm resolution and a scanning speed of 20 s in a quartz cuvette with a 1 mm path length.

### Protease Resistance Assay

Proteolysis was measured by RP-HPLC using the following parameters, as reported.^33^ Trypsin with a final concentration of 10 μg/mL was added to a solution of the peptides in PBS (pH 7.4) (100 μM). The reaction was monitored over time using reversed-phase HPLC (C18 reversed-phase Bio-Rad analytical column, 250 × 4 mm, 300 Å pore size, and 7 μm particle size). The column was eluted in 40 min using a linear gradient of 10–90% acetonitrile in water, both containing 0.1% trifluoroacetic acid (v/v), at a flow rate of 0.6 mL/min.

### Plasma Stability Testing

The stability of peptides in human blood plasma was measured using a literature procedure.^10,34^ Plasma was separated from red blood cells over centrifugation, frozen, and stored at −80 °C until use. Briefly, peptides were prepared as a 1 mM solution in PBS (pH 7.4). In 80 μL of blood plasma (20% v/v) was diluted 20 μL of the peptide solution. The solution was incubated at 37 °C for different time points and then added to 100 μL of a mixture containing 80% acetonitrile, 10% methanol. amd 10% water to stop the further degradation of the peptides. The cloudy solution was produced upon the addition of the stopping solution. This solution was cooled to 4 °C for 1 h and then centrifuged at 10 000 rpm for 10 min to remove the plasma proteins as a residue. The supernatant (50 μL) was injected onto a reversed-phase HPLC (C18 reversed-phase Bio-Rad analytical column, 250 × 4 mm, 300 Å pore size, and 7 μm particle size). The column was eluted in 40 min using a linear gradient of 10–90% acetonitrile in water, both containing 0.1% TFA (v/v), at a flow rate of 0.6 mL/min, and the absorbance was detected at 215 nm. The decrease in the chromatographic peak area determined the percentage of remaining peptide in each sample.

### Antimicrobial Assays

For our experiments, we used strains obtained from the American Type Culture Collection (ATCC), including *P. aeruginosa* PAO1, *E. coli* K12 parental type, *S. aureus* ATCC 6538P, and *B. subtilis* ATCC 6051. The MIC assays were performed as reported by Wiegand et al. using the broth microdilution method in 96-well round-bottom microplates.^35^ The MIC was defined as the lowest concentration at which no bacterial growth was observed. Briefly, bacteria were inoculated into Mueller Hinton broth (MHB), cultured overnight at 37 °C, and transferred to a new MHB until the exponential phase of growth. The bacteria were diluted to 1 × 10^6^ CFU/mL using MHB, and the peptides were prepared in double-distilled water (DDW) filtered over a 0.25 μm filter. To 50 μL of the MHB medium containing two fold serially diluted peptides with concentrations ranging from 1.56 to 50 μM were added 50 μL aliquots of the bacterial solution. The plates were incubated for 24 h at 37 °C. The positive and negative controls used were cultured without peptides (100% growth) and uninoculated MHB broth (0% growth). The bacterial growth inhibition was evaluated by measuring the absorbance at 600 nm using a microplate autoreader (SynersyMx, Biotek).

### Flow Cytometry Analysis

The effect of the peptides on the bacterial membrane was evaluated using a Stratedigm S1000EON flow cytometer and analyzed by using CellCapTure software (San Jose, CA). SYTOX-Green (Sigma-Aldrich) is a DNA binding dye that only labels cells with compromised membranes.^36^ FM 4-64 is a lipophilic fluorescent dye. SYTOX-Green and FM 4-64 were excited with the cyan 488 nm laser. Forward scatter (FSC) and side scatter (SSC) as well as green (530/30 nm filter) and red (710/40 nm filter) fluorescence emissions were measured. Briefly, the experiments were done in three steps. (1) A fresh MHB culture of bacteria was diluted to a final concentration of 10^6^ cells/mL in 10 mM sodium phosphate buffer (pH 7.4). (2) Lipophilic FM 4-64 (1 μM/mL) was used to label the cells, followed by the addition of SYTOX-Green to the same bacterial suspension with the final concentration of 1 μM/mL. (3) The peptides at their MIC concentrations were added to the bacteria, FM 4-64, and SYTOX-Green suspension. The samples were tested in the flow cytometer immediately after each step. The temporal effect of the peptide on the permeability of the bacteria membrane was measured.

### Fluorescence Confocal Microscopy Imaging

FM 4-64 (1 μM/mL) fluorescent dye was used to label the bacterial cells, and SYTOX-Green (1 μM/mL) was used to evaluate the cell membrane disruption. In brief, a fresh MHB culture of bacterial cells was diluted to a final concentration of 1 × 10^6^ cells/mL in 10 mM sodium phosphate buffer (pH 7.4), followed by the addition of FM 4-64 and SYTOX-Green, and were then treated with the peptides at their MIC concentrations. Finally, 15 μL of the bacterial suspension was spread on a glass slide. A thin layer of an agarose pad was used to fix the bacterial cells, and cells examined using an Olympus IX81 FV10-ASW fluorescence confocal microscope with an objective of X60 (oil) NA:1.35. SYTOX-Green was observed with excitation and emission wavelengths of 488 and 500–545 nm, respectively, and FM 4-64 dye was observed with excitation and emission wavelengths of 559 and 655–755 nm.

## Supporting Information Available

HPLC chromatograms of all peptides, mass spectra of Amp1L and AMP1EP9, and flow cytometry results for all bacteria (PDF)

## Abbreviations Used

AMP: antimicrobial peptides
MDR: multidrug-resistant
CD: circular dichroism
MIC: minimum inhibitory concentration
DIC: *N*,*N*′-diisopropylcarbodiimide
oxyma: ethyl cyanohydroxyiminoacetate
TFA: trifluoroacetic acid
TIS: triisopropyl silane
DMF: *N*,*N*-dimethylformamide
DCM: dichloromethane
RP-HPLC: reversed-phase high-performance liquid chromatography
hRBCs: human red blood cells
RAW 264.7: murine macrophage cells
HEK 293: human embryonic kidney cells
XTT: 2,3-bis(2-methoxy-4-nitro-5-sulfophenyl)-2*H*-tetrazolium-5-carboxanilide
PBS: phosphate-buffered saline
HEPES: (4-(2-hydroxyethyl)-1-piperazineethanesulfonic acid
LPS: lipopolysaccharide
MHB: mueller Hinton broth
CFU: colony-forming unit
DDW: double-distilled water
ATCC: American Type Culture Collection
