# Supplementary material for: Site-Specific Isopeptide Bond Formation: A Powerful Tool for the Generation of Potent and Nontoxic Antimicrobial Peptides
Source: J Med Chem. 2022 Mar 15;65(6):5085–94. doi: 10.1021/acs.jmedchem.2c00061 (PMC8958506; doi:10.1021/acs.jmedchem.2c00061)
Supplement: Supplementary file 1 — jm2c00061_si_001.pdf [file jm2c00061_si_001.pdf]

Supporting Information

Site-specific Isopeptide Bond Formation: a Powerful Tool for the Generation of Potent and Nontoxic Antimicrobial Peptides

Naiem Ahmad Wani, Elad Stolovicki, Daniel Ben Hur, Yechiel Shai*

Department of Biomolecular Sciences, Weizmann Institute of Science, Rehovot 76100, Israel.

* Corresponding author

Email: Yechiel.Shai@weizmann.ac.il

Contents:

Figure S1: Analytical HPLC chromatograms of peptides

Figure S2: Mass spectrum of peptide Amp1L

Figure S3: Mass spectrum of peptides Amp1EP9

Figure S4: Flow cytometry results containing scatter plots and labelling of FM4-64 dye on *P. aeruginosa*, *E.coli*, *S. aureus* and *B. subtilis* treated with peptides Amp1L and Amp1EP9

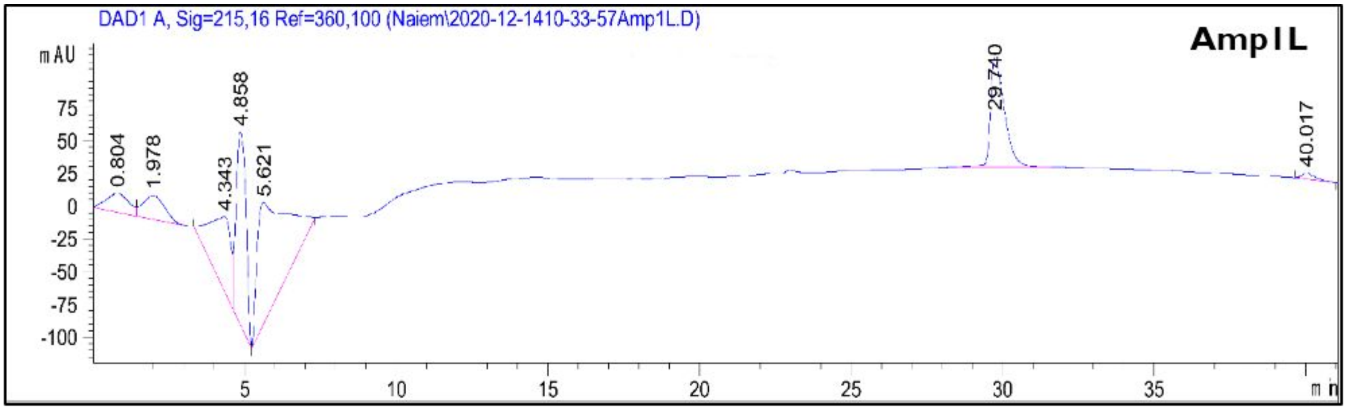
DAD1 A, Sig=215,16 Ref=360,100 (Naiem\2020-12-1410-33-57Amp1L.D)
Amp1L
mAU
0.804
1.978
4.343
4.858
5.621
29.740
40.017
min

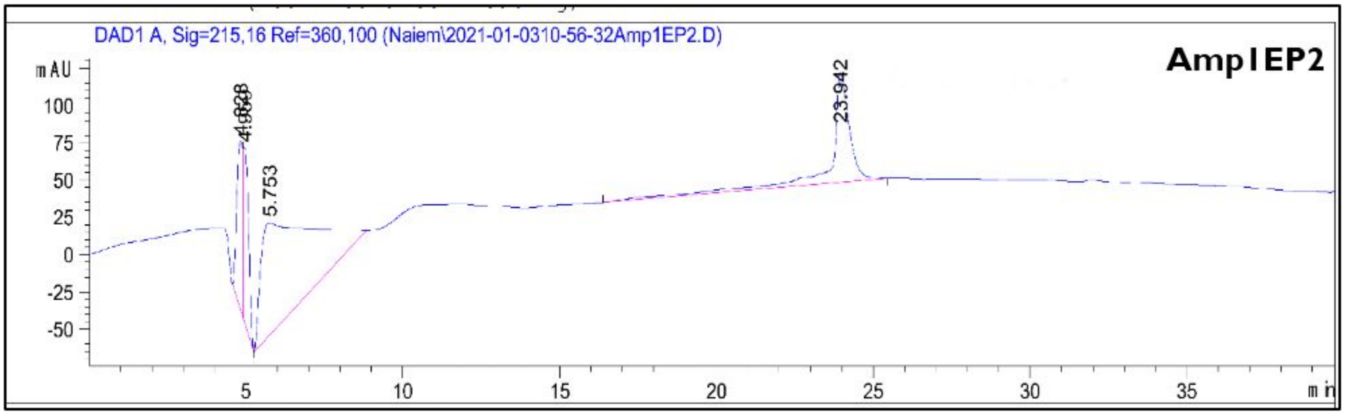
DAD1 A, Sig=215,16 Ref=360,100 (Naiem\2021-01-0310-56-32Amp1EP2.D)
Amp1EP2
mAU
5.753
23.942
min

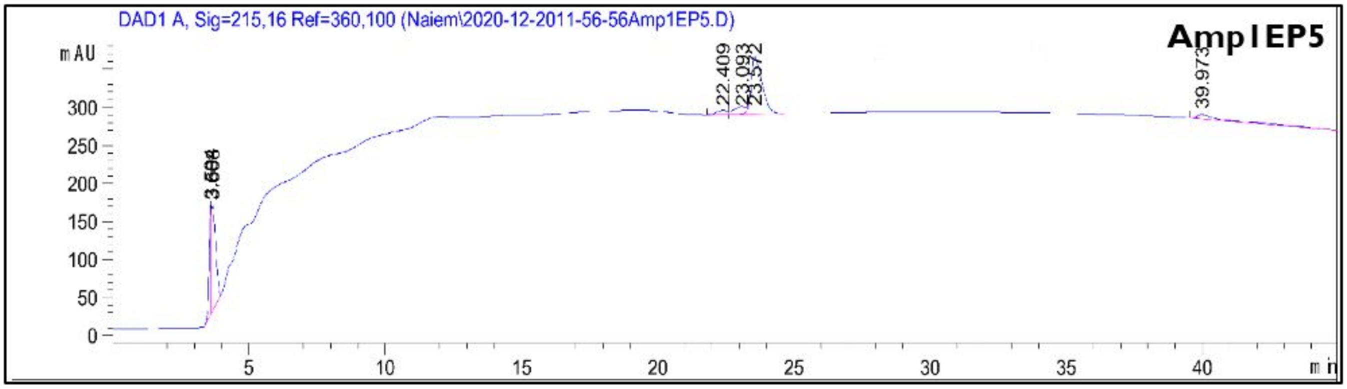
DAD1 A, Sig=215,16 Ref=360,100 (Naiem\2020-12-2011-56-56Amp1EP5.D)
Amp1EP5
mAU
22.409
23.093
23.572
39.973
min

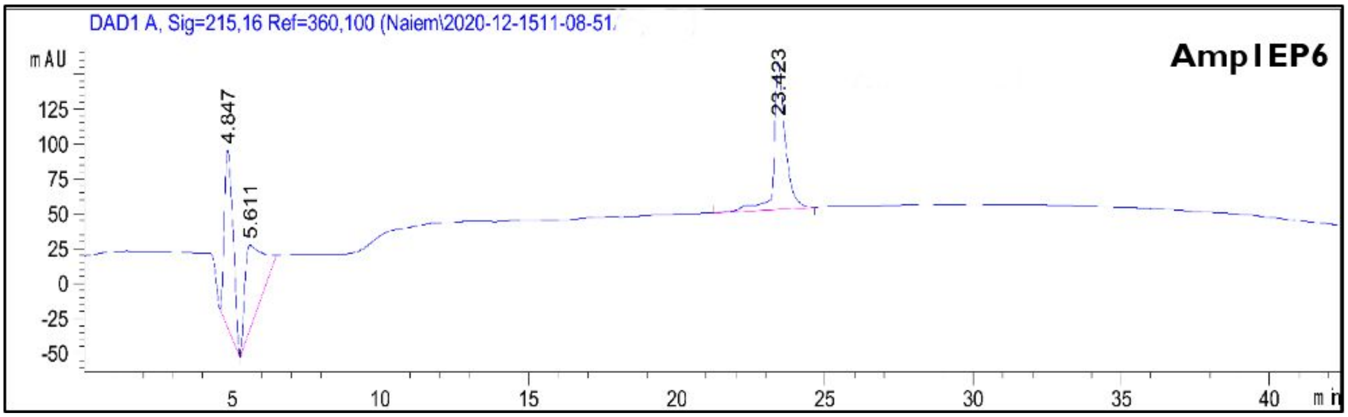
DAD1 A, Sig=215,16 Ref=360,100 (Naiem\2020-12-1511-08-51
Amp1EP6
mAU
4.847
5.611
23.423
min

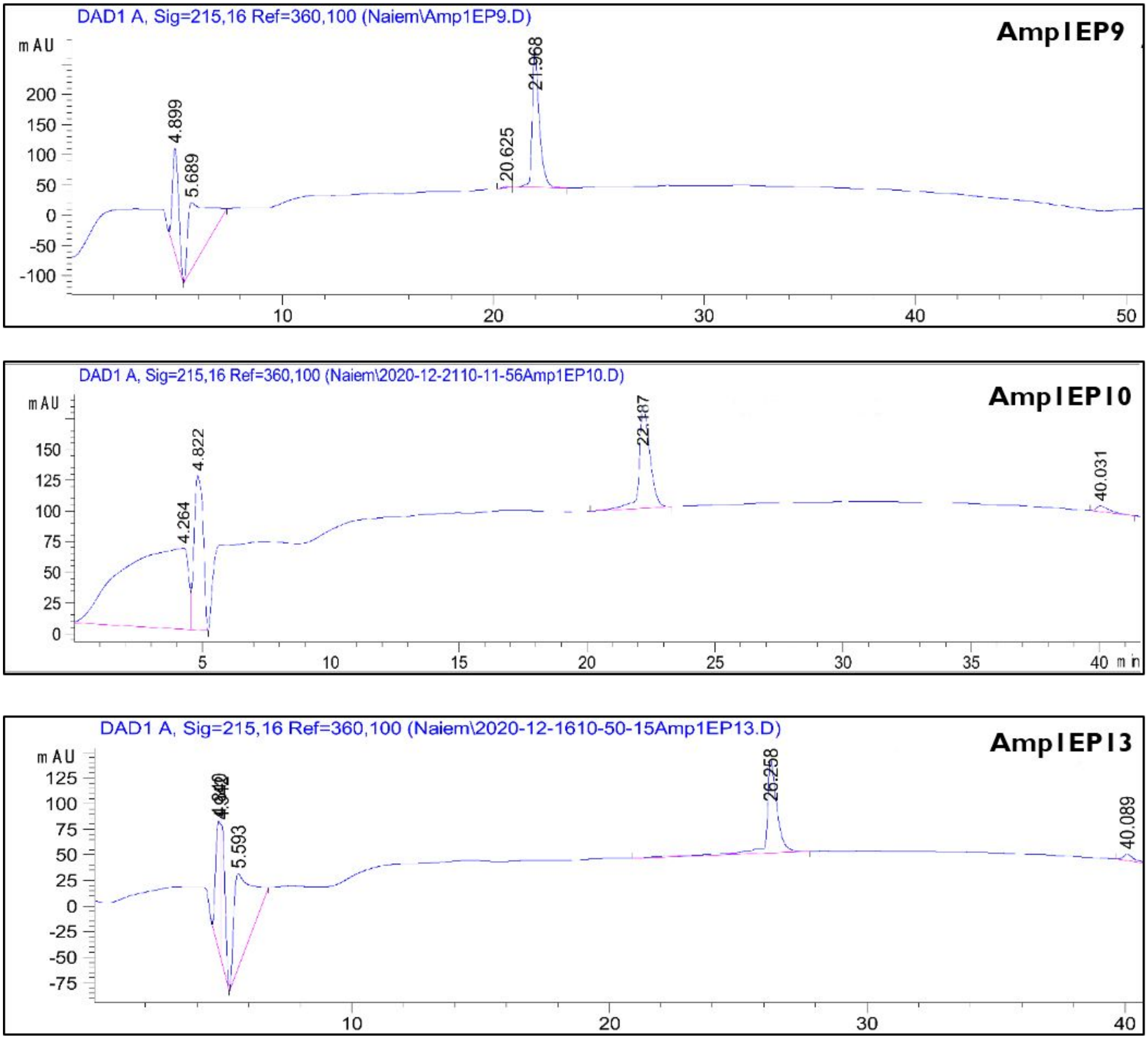

Figure S1: HPLC chromatograms of peptides

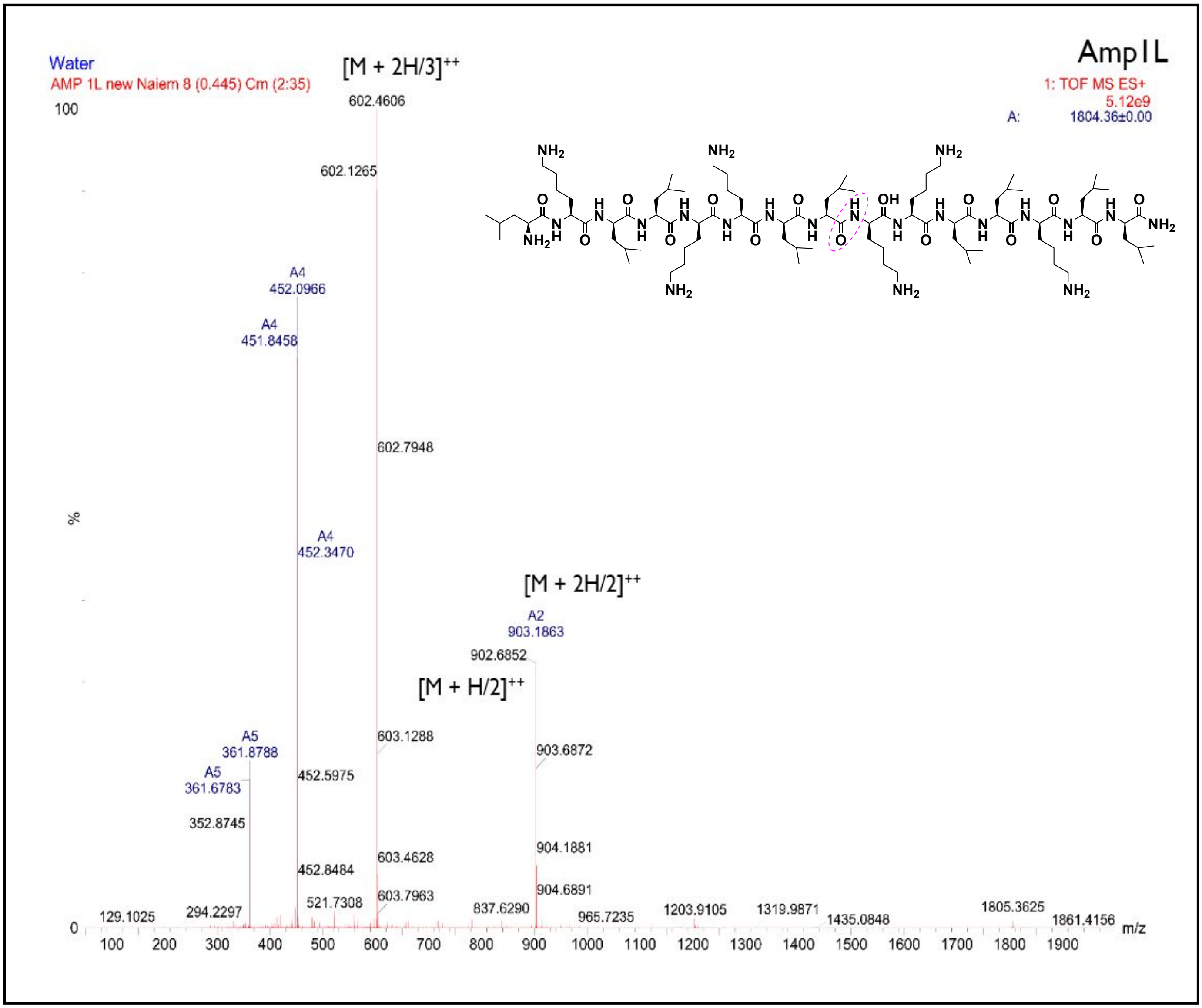

Figure S2: Mass spectra of peptide Amp1L

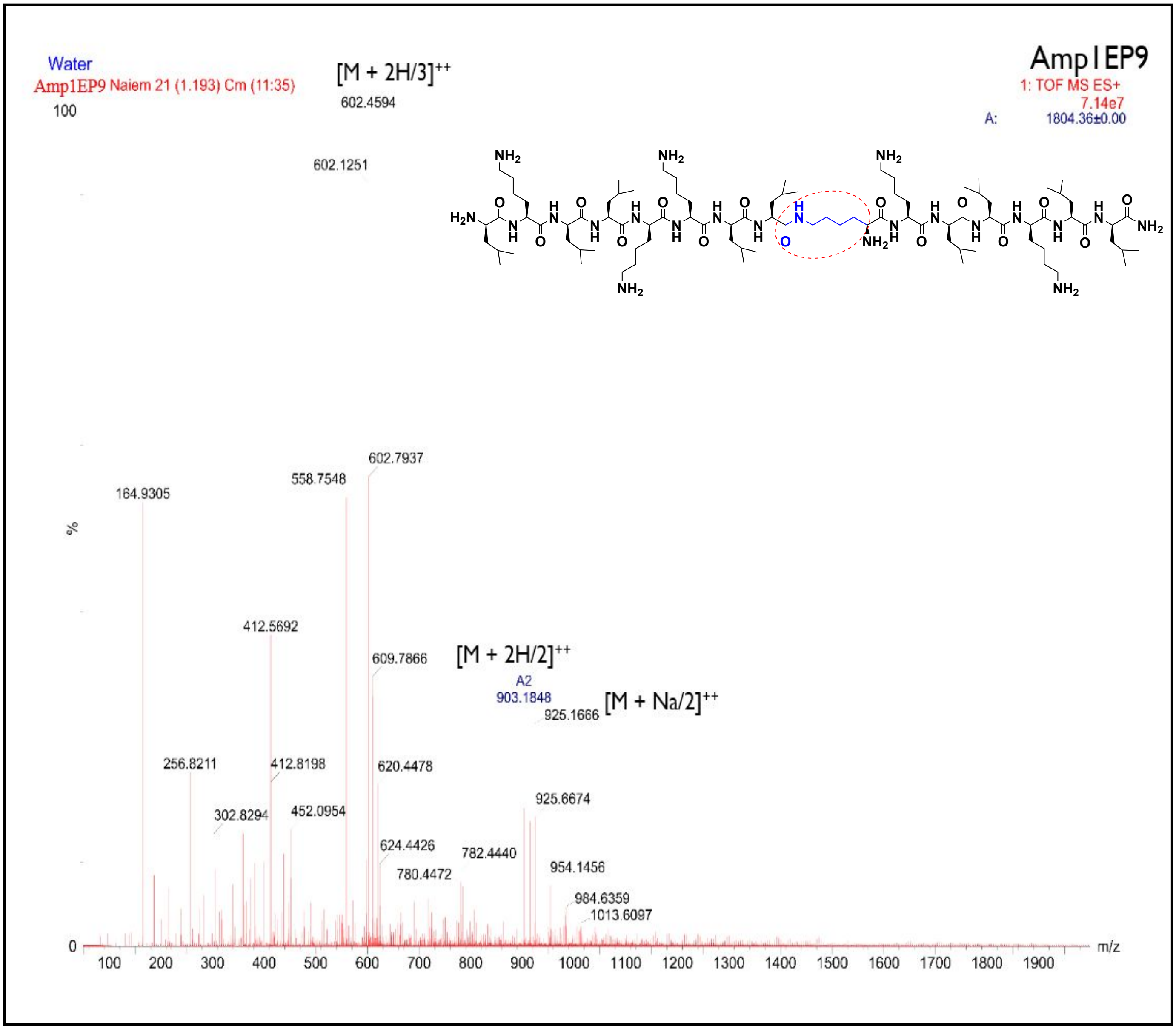

Figure S3: Mass spectra of peptide Amp1EP9

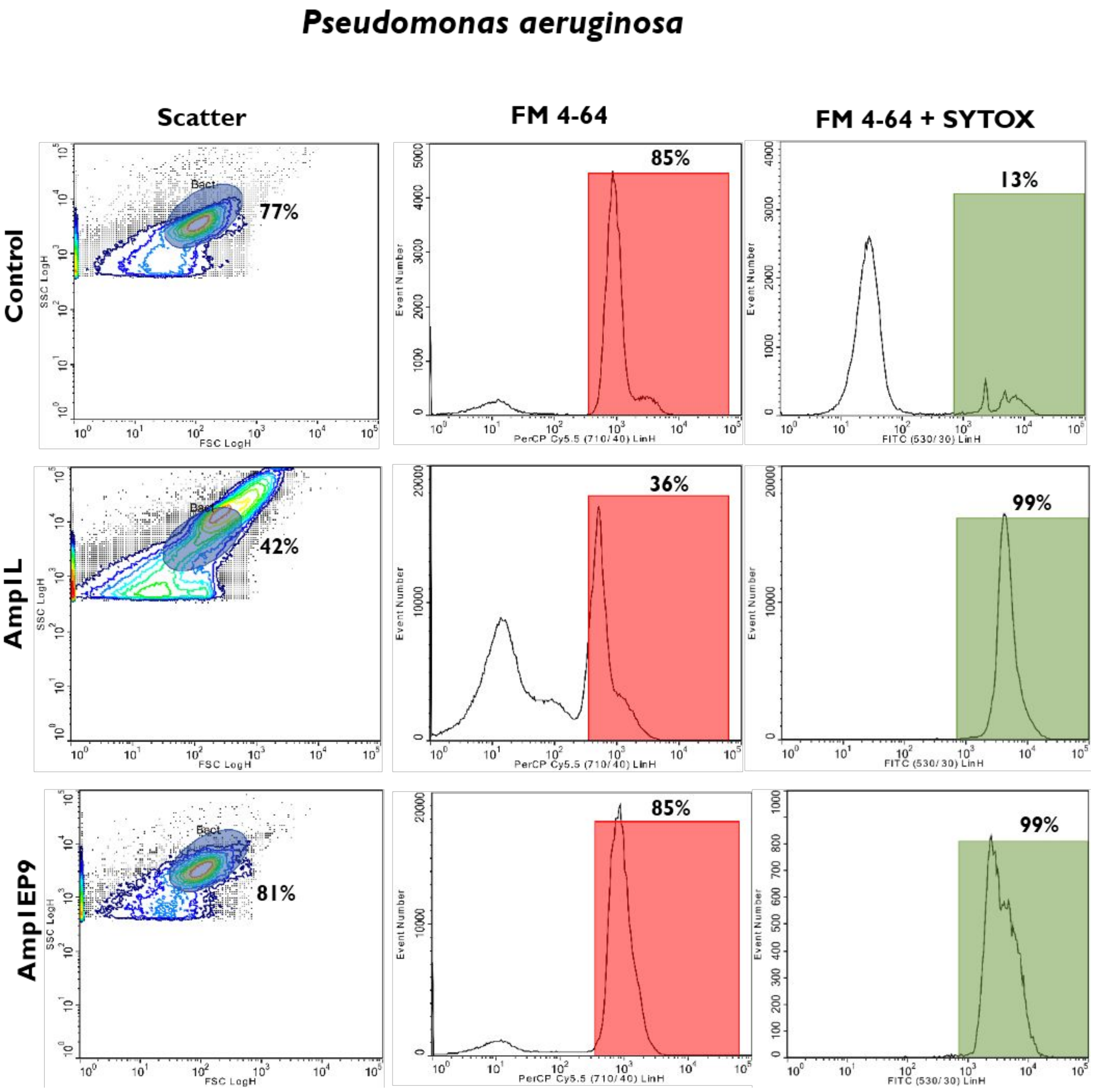
Pseudomonas aeruginosa
Scatter
FM 4-64
FM 4-64 + SYTOX
Control
Amp1L
Amp1EP9
77%
85%
13%
42%
36%
99%
81%
85%
99%
Bact
SSC LogH
FSC LogH
Event Number
PerCP Cy5.5 (710/40) LinH
FITC (530/30) LinH

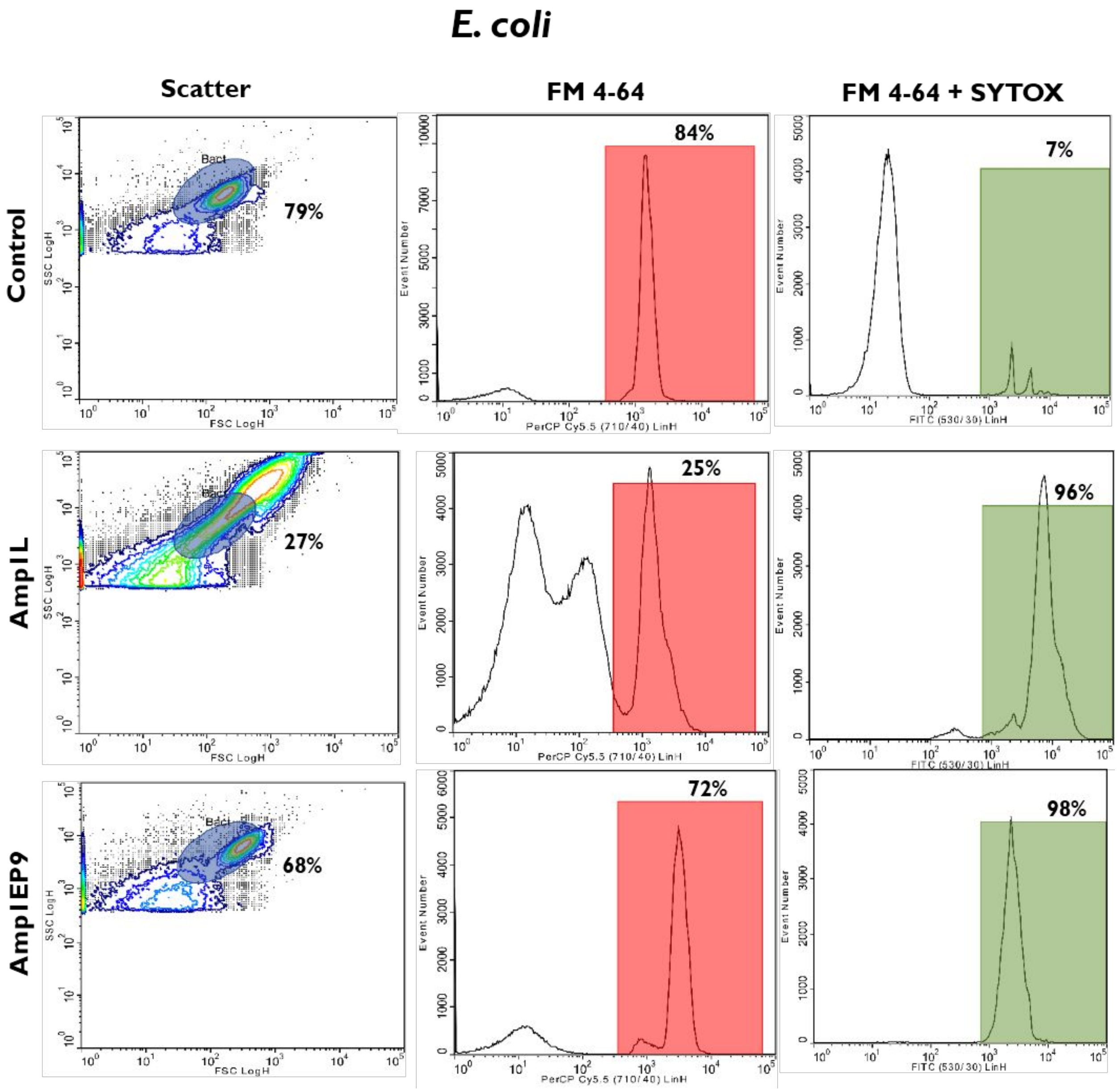
E. coli
Scatter
FM 4-64
FM 4-64 + SYTOX
Control
Amp1L
Amp1EP9
79%
84%
7%
27%
25%
96%
68%
72%
98%
Bact
SSC LogH
FSC LogH
Event Number
PerCP Cy5.5 (710/40) LinH
FITC (530/30) LinH

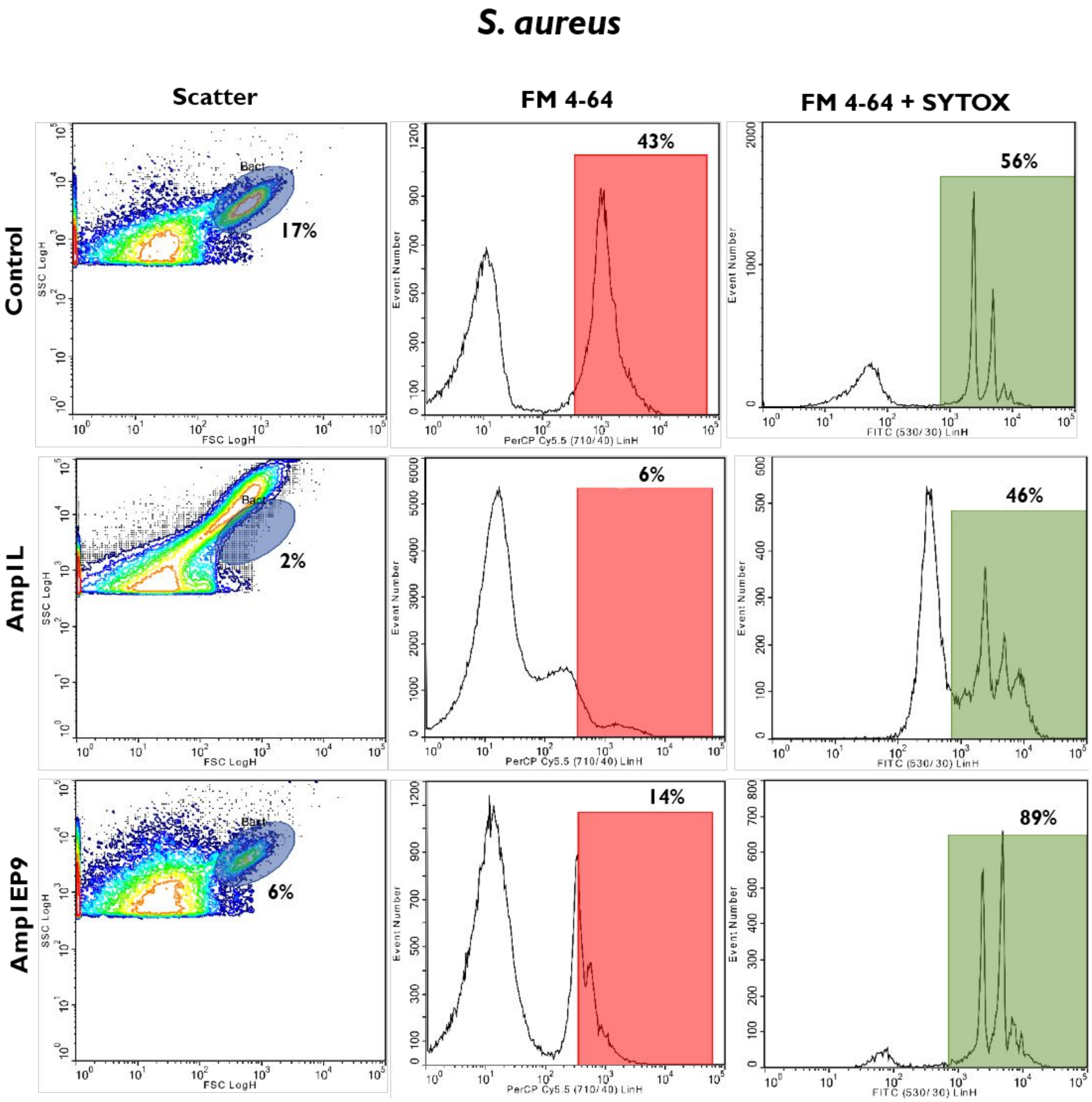
S. aureus
Scatter
FM 4-64
FM 4-64 + SYTOX
Control
Amp1L
Amp1EP9
17%
2%
6%
43%
6%
14%
56%
46%
89%
Bact
SSC LogH
FSC LogH
Event Number
PerCP Cy5.5 (710/40) LinH
FITC (530/30) LinH

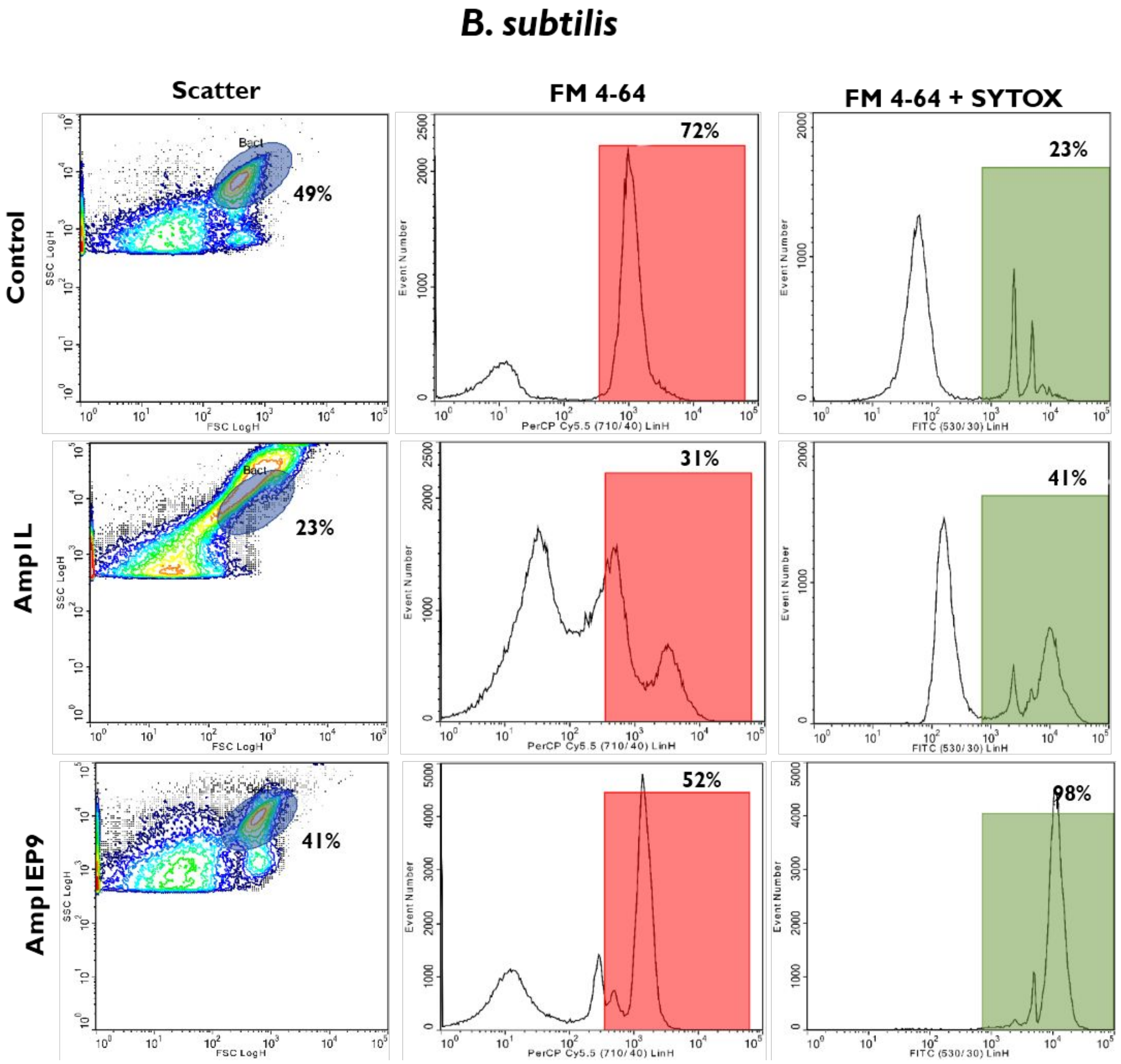

Figure S4: Flow cytometry results of membrane permeability experiments of *P. aeruginosa*, *E.coli*, *S. aureus* and *B. subtilis* treated with peptides Amp1L and Amp1EP9. Side scatter (SCC) vs. forward scatter (FSC) (left column). Note that treatment with Amp1L shift the scattering pattern of the bacteria out of the "Bact" gate (blue oval shape). All bacteria cells should be labeled with the membrane dye FM 4-64 (center column). Cells with compromised membrane are labeled with SYTOX green. Only events that are red positive are considered bacteria and are plotted in the histograms (right column).
